# Mitochondrial DNA genomes of five major *Helicoverpa* pest species from the Old and New Worlds (Lepidoptera: Noctuidae)

**DOI:** 10.1002/ece3.4971

**Published:** 2019-02-14

**Authors:** Tom K. Walsh, Omaththage Perera, Craig Anderson, Karl Gordon, Cecilia Czepak, Angela McGaughran, Andreas Zwick, Daniel Hackett, Wee Tek Tay

**Affiliations:** ^1^ CSIRO Black Mountain Laboratories Canberra ACT Australia; ^2^ USDA‐ARS Southern Insect Management Research Unit Stoneville Mississippi; ^3^ MRC Human Genetics Unit, MRC Institute of Genetics and Molecular Medicine, Western General Hospital University of Edinburgh Edinburgh UK; ^4^ Escola de Agronomia Universidade Federal de Goiás Goiânia Brazil; ^5^ Division of Ecology and Evolution, Research School of Biology Australian National University Canberra ACT Australia; ^6^ London UK

**Keywords:** biosecurity, COI, lepidopteran pests, mitogenomes

## Abstract

Five species of noctuid moths, *Helicoverpa armigera*, *H. punctigera*, *H. assulta*, *H. zea,* and *H. gelotopoeon,* are major agricultural pests inhabiting various and often overlapping global distributions. Visual identification of these species requires a great deal of expertise and misidentification can have repercussions for pest management and agricultural biosecurity. Here, we report on the complete mitochondrial genomes of *H. assulta assulta* and *H. assulta afra*, *H. gelotopoeon, H. punctigera, H. zea*, and* H. armigera armigera* and *H. armigera conferta’* assembled from high‐throughput sequencing data. This study significantly increases the mitogenome resources for these five agricultural pests with sequences assembled from across different continents, including an *H. armigera* individual collected from an invasive population in Brazil. We infer the phylogenetic relationships of these five *Helicoverpa* species based on the 13 mitochondrial DNA protein‐coding genes (PCG's) and show that two publicly available mitogenomes of *H. assulta* (KP015198 and KR149448) have been misidentified or incorrectly assembled. We further consolidate existing PCR‐RFLP methods to cover all five *Helicoverpa* pest species, providing an updated method that will contribute to species differentiation and to future monitoring efforts of *Helicoverpa* pest species across different continents. We discuss the value of *Helicoverpa* mitogenomes to assist with species identification in view of the context of the rapid spread of *H. armigera* in the New World. With this work, we provide the molecular resources necessary for future studies of the evolutionary history and ecology of these species.

## 1 INTRODUCTION

Accurate species identification is the foundation for all biological research; however, the scientific community is often distracted by polarized support either for traditional morphological or for molecular identification of species (e.g., Hebert, Penton, Burns, Janzen, & Hallwachs, 2004; Rubinoff, 2006). However, it is also becoming increasingly clear that both methods contribute value and should be better integrated to provide stronger support for defining species status (e.g., Desalle, 2006). Confusion in the scientific literature, especially relating to visually similar organisms, can lead to substantial difficulty in formulating and developing management, trade, and economic policies. Furthermore, the availability of high‐throughput sequencing data is revealing that hybridization between so‐called species, is perhaps more common than was previously thought (Anderson et al., 2018; Anderson, Tay, McGaughran, Gordon, & Walsh, 2016; Elfekih et al., 2018).

Examples of this conflict between molecular and morphological identification include the stored grain beetle, *Cryptolestes* spp., where despite recent studies combining molecular data and morphology, confusion remains (Tay, Beckett, & De Barro, 2016; Wang et al., 2014). In contrast, a successful example of integrating DNA data, with morphological and phenotypic characters to differentiate species, is the differentiation Asian and European honeybee mite species, *Varroa jacobsoni* and* V. destructor*, respectively (Anderson & Trueman, 2000).

Confident and unambiguous identification of invasive organisms especially those with agricultural and economic significance is becoming increasingly important in a highly mobile world. This can be seen with the recent incursion of the Old World cotton bollworm, *Helicoverpa armigera*, into the New World (e.g., Czepak, Albernaz, Vivan, Guimarães, & Carvalhais, 2013; Tay et al., 2013), and the detection of both sister species of the fall army worm (FAW), *Spodoptera frugiperda*, in Africa (Cock, Beseh, Buddie, Cafa, & Crozier, 2017; Goergen, Kumar, Sankung, Togola, & Tamò, 2016; Nagoshi et al., 2017; Otim et al., 2018). Although the timing of *S. frugiperda*'s arrival to the African continent is as yet unknown, the arrival of *H. armigera* in Brazil occurred sometime before the cropping season of 2012/13 when it was first identified from historical sampling efforts (Sosa‐Gómez et al., 2015). The morphological similarity between *H. armigera* and the New World *H. zea* was likely an important factor for the delay in detection. Various studies (Anderson et al., 2018, 2016; Arnemann, 2015; Arnemann et al., 2016; Arneodo, Balbi, Flores, & Sciocco‐Cap, 2015; Mastrangelo et al., 2014; Tay et al., 2013) have shown that *H. armigera* populations in Brazil and neighboring countries had wide potential geographic origins from Asia, Africa, and Europe, with their introductions having a strong association with global agricultural and horticultural trade movements into South America (Tay, Walsh et al., 2017).

Co‐occurring with *H. armigera* across the Old World is the Solanaceae specialist *H. assulta*, while *H. punctigera*, a major agricultural pest in itself, is endemic to Australia (for a review see Hardwick, 1965). *Helicoverpa armigera*, *H. punctigera,* and *H. zea* are morphologically similar and identifying them has traditionally relied on dissecting the adult male and female genitalia (e.g., Hardwick, 1965; Pogue, 2004), which is both time consuming and technically challenging. Studies by Behere, Tay, Russell, and Batterham (2008) and Fang et al. (1997) have previously assessed mtDNA and nuclear DNA genes to distinguish between the major *Helicoverpa* pest species. Behere et al. (2008) developed a PCR‐RFLP method of identifying *H. armigera*, *H. zea*, *H. punctigera,* and *H. assulta* based on partial mtDNA COI and cytochrome *b* (Cytb) genes. Arneodo et al. (2015) applied the concept of Behere et al. (2008) and developed a RFLP method to assist with the rapid differentiation between New World *H. zea* and *H. gelotopoeon* and *H. armigera*. However, both Behere et al. (2008) and Arneodo et al. (2015) used different mtDNA COI gene regions, and identification by PCR‐RFLP between these five *Helicoverpa* pest species would therefore require different PCR amplicons.

Recent studies relating to the molecular characterization of complete mitochondrial DNA genomes (mitogenomes) have used high‐throughput sequencing technology that enables rapid mitogenome assembly of a wide range of insect species. High‐throughput sequencing platforms with improved bioinformatic pipelines for assembling mitogenomes have also been shown to be an ideal option for studying historical specimens, in vertebrates (e.g., Anmarkrud & Lifjeld, 2017) as well as insects (e.g., Tay, Elfekih et al., 2017), where genomic DNA is typically fragmented due to the age of samples, and/or poor preservation conditions. These factors represent a significant challenge to the Sanger method (Sanger & Coulson, 1975) of sequencing PCR amplicons. Furthermore, applying high‐throughput sequencing methods also bypasses potential primer annealing issues, gDNA template limitations, and reduces the chances and impact of contamination.

Currently, there are published mitogenomes of *H. armigera* (Yin, Hong, Wang, Cao, & Wei, 2010) from China, *H. punctigera* (Walsh, 2016) from Australia, *H. zea* (Perera, Walsh, & Luttrell, 2016) from the United States of America, two mitogenomes of *H. assulta* from China, but no mitogenomes for *H. gelotopoeon*. For the *H. assulta* mitogenomes, one has been published (Li, Zhang, Luo, Cui, & Dong, 2016; GenBank KP015198), while the second is unpublished but publicly available (GenBank KR149448). In this study, we report on the molecular characterization of an additional 15 mitogenomes that include the New World *H. gelotopoeon* from Argentina and a Brazilian *H. zea, *the Old World *H. assulta* (subspecies *assulta*, present in Asia, Australasia, Europe) and subspecies *afra* (present in Africa, south of the Sahara Desert); (Hardwick, 1965), *H. armigera conferta,* (present in Australia) and *H. armigera armigera* (present in Asia, Europe, Africa (Hardwick, 1965) and South America (Anderson et al., 2018, 2016)), and increase the mitogenome resources of the Australian endemic *H. punctigera *(Table 1). We show that the available *H. assulta* mitogenomes are affected by misidentification (KP015198) and sequencing errors (KR149448). We also consolidate the current PCR‐RFLP methods for species identification to include all five *Helicoverpa* pest species. Furthermore, we discuss the biosecurity implications of our study with respect to pest species identification and the importance of accurately characterized mitogenomes, while providing the molecular resources necessary for future studies of the evolutionary history and ecology of these *Helicoverpa* pest species.

**Table 1:** Samples and sequences used in this work. *Helicoverpa geletopoeon* (*n* = 2), *H.* *assulta* *assulta* (*n* = 2), *H.* *assulta* *afra* (*n* = 1), *H.* *punctigera* (*n* = 2), *H.* *armigera* *conferta* (*n* = 2), *H.* *armigera* *armigera* (*n* = 5), and *H.* *zea* (*n* = 1) draft mitogenomes (mtGenome) lengths, collection dates of individuals, country of origins, and GenBank accession numbers assembled for this study

*Helicoverpa* sp.	Draft mitogenome length (bp)	Collection date	Country	GenBank
*gelotopoeon*	15,226	2013	Argentina	MG437199
*gelotopoeon*	15,230	2013	Argentina	MG437189
*assulta assulta*	15,184	2013	Australia	MG437197
*assulta assulta*	15,400	1986	Thailand	KT626655
*assulta afra*	15,403	1981	Tanzania	MG437198
*punctigera*	15,382	2013	Australia	KF977797
*punctigera*	15,382	2013	Australia	MG437200
*punctigera*	15,374	2013	Australia	MG437201
*armigera armigera*	15,347	NA	China	GU188273
*armigera conferta*	15,347	2013	Australia	MG437194
*assulta* (***armigera*** a)	15,351	NA	China	KP015198
*armigera conferta*	15,311	2013	Australia	MG437193
*armigera armigera*	15,344	2013	Brazil	MG437190
*armigera armigera*	15,234	2013	Spain	MG437191
*armigera armigera*	15,249	2005	Uganda	MG437196
*armigera armigera*	15,322	2004	India	MG437192
*armigera armigera*	15,373	2006	Madagascar	MG437195
*zea*	15,343	2014	USA	KJ930516
*zea*	15,352	2012	Brazil (Hz073)	MG437202

a This *Helicoverpa assulta* individual (KP015198) is highly likely to be a misidentified *H. armigera armigera* individual from China based on nucleotide sequence identity and phylogenetic analysis as presented in this current study.

## 2 MATERIAL AND METHODS

### 2.1 *Helicoverpa species *DNA library construction and sequencing

Fifteen mitogenomes were sequenced in this work: *H. assulta assulta* (*n* = 2), *H. assulta afra *(*n* = 1), *H. gelotopoeon* (*n* = 2), *H. zea* (*n* = 1), *H. punctigera* (*n* = 2), *H. armigera conferta* (*n* = 2), and *H. armigera armigera* (*n* = 5). The remaining preserved material and DNA is stored and available from the authors upon request. The *H.* *assulta* individual (I.D.343) was collected from Northern NSW, Australia in 2013. The second (KT626655) was from a sample collected in Thailand in 1986 that was identified and preserved as a pinned reference specimen by the late Dr. Marion Laster (Entomologist, Southern Insect Management research Unit, Stoneville, MS, USA). The third was a historical pinned *H.* *assulta* *afra *specimen from Tanzania (November 1981 by DH). Two *H.* *gelotopoeon* individuals were from San Miguel de Tucuman state in Argentina and were collected in 2013 from a light trap (Table 1).

With the exception of the pinned historical *H.* *assulta* *afra* from Tanzania and *H.* *assulta* *assulta* from Thailand, all *Helicoverpa* specimens were stored in ≥95% ethanol. DNA was purified using the Blood and Tissue DNA extraction kit (Qiagen), prior to quantification using Qubit (Life Technologies). Sequencing libraries were constructed as reported in Walsh (2016). DNA extraction from *H.* *assulta* *assulta* (KT626655) was as reported in Perera et al. (2015), and the DNA library was constructed as described in Perera et al. (2016). DNA library sequencing was performed at the Australian National University Biomolecular Resource Facility (Canberra, Australia) and the USDA‐ARS Genomics and Bioinformatics Research Unit, (Stoneville, MS, USA).

Initial identification of adult *H.* *gelotopoeon* specimens from *H.* *zea*/*H.* *armigera* was as described by Hardwick (1965) using the adult morphology and subsequently confirmed by partial mtCOI sequence identity prior to complete mtDNA genome characterization. For the differentiation between subspecies of *H.* *assulta,* we followed the guidelines outlined by (for a review see Hardwick, 1965); that is, *H.* *a.* *assulta* present in Asia, Australasia, Europe; *H.* *a.* *afra* present in Africa, south of the Sahara Desert.

### 2.2 Mitogenome assembly

For the assembly of the *Helicoverpa* mitogenomes (Table 1), we used two separate methods involving different assembly programs of either the genomic software Geneious R8 version 8.1.9 (Biomatters Pty Ltd., Auckland, NZ) or the CLC Genomic Workbench v8.5 (Qiagen). The mitogenome of *H.* *assulta *from Thailand was de novo assembled using CLC Genome Workbench, and the remainder of the mitogenomes was assembled using mitogenomes of *H.* *punctigera *(KF977797), *H.* *armigera* (GU188273), and *H.* *zea* (KJ930516) as reference sequences. After the initial sequence assembly based on the appropriate reference mitogenome, we reassembled these mitogenomes against their first version mitogenome templates. With each subsequent reassembly, we fine‐tuned and removed all ambiguity by manually checking for potential misassembled regions. This procedure was repeated between three to eight times until a complete draft mitogenome was obtained. Draft mitogenomes from this study are available in GenBank (MG437189‐MG437202, KT626655; Table 1).

### 2.3 Molecular characterization of *Helicoverpa* draft mitogenomes

To characterize the assembled draft mitogenomes of all *Helicoverpa* species, we used the program MITOS (Bernt et al., 2013), specifying the invertebrate genetic code (code #5) for identifying all tRNAs, rRNAs, and the start of protein‐coding genes (PCGs). The origin of replication in our assembled mitogenomes was putatively identified, and due to its low complexity nature, we inserted a string of 5N's to indicate potential assembly difficulty across this region. The characterized mitogenomes were manually adjusted for stop codons to indicate the end of the PCGs using the published *H.* *zea* mitogenome PCGs as reference (Perera et al., 2016, KJ930516), although we note that to identify the most likely stop codon would require sequencing of RNA reverse transcribed cDNA (Gissi & Pesole, 2003).

### 2.4 Confirmation of species identity

We examined all mitogenome PCGs using BlastN (Altschul et al., 1997) to confirm species identity. To confirm our putative *H.* *assulta* *assulta *individuals (MG437197; KT626655), we compared our *H.* *assulta* *assulta* PCGs against both the published (Li et al., 2016; KP015198 and NC_026199) and unpublished *H.* *assulta* mitogenome (GenBank KR149448). The published mitogenome (KP015198) was reported from an individual collected from a cotton host in Anyang (Henan Province, China). We aligned our newly assembled *H.* *assulta* mitogenomes and those available in GenBank using the nucleotide alignment program MAFFT v7.017 (Katoh, Misawa, Kuma, & Miyata, 2002), implementing default settings (i.e., automatic algorithm option, Scoring Matrix: 200PAM/K2, Gap open penalty 1.53; Offset value: 0.123) within Geneious v8.1.9. Due to the significant differences detected between our two *H.* *assulta* mitogenomes (MG437197, KT626655.1) and the previously reported mitogenomes (GenBank KR149448, NC_026199/KP015198) from both MAFFT alignment and BlastN searches, we realigned these four *H.* *assulta* mitogenomes against the published *H.* *armigera* mitogenome available in GenBank (GU188273; Yin et al., 2010).

### 2.5 Phylogenetic analysis

We performed a phylogenetic analysis using all 13 protein‐coding genes (PCGs) found in the publicly available mitogenomes of selected noctuid species: (a) *Agrotis segetum* (KC894725, Wu, Cui, Du, Gu, & Wei, 2014), (b) *A.* *ipsilon* (KF163965, Wu, Cui, & Wei, 2015), (c) *Spodoptera frugiperda* (KM362176, (Liu, Chai et al., 2016), (d) *S.* *litura* (JQ647918, Wan, Kim, & Kim, 2013); (KF543065, Liu, Zhu et al., 2016), (e) *Chloridea* (*Heliothis*) *subflexa* (KT598688, de Souza, Tay, Czepak, Elfekih, & Walsh, 2016), selected available *Helicoverpa* species mitogenomes that included (f) *H.* *punctigera* (KF977797, Walsh, 2016), *H.* *assulta* *assulta* (KP015198, Li et al., 2016); KR149448, (unpublished), *H.* *zea* (KJ930516, Perera et al., 2016), and *H.* *armigera* *armigera *(GU188273, Yin et al., 2010). All complete mitogenome sequences were first aligned using the MAFFT alignment program with default parameters as detailed above. Next, all PCG's were readjusted to include a stop codon, while the start codon was as determined by MITOS (Bernt et al., 2013), prior to extraction to Geneious R8 for fine‐scale alignment and sequence trimming where necessary (Supporting Information Data S1: Aligned PCGs). Phylogenetic analysis of the concatenated PCGs from all noctuid species was carried out using the PhyML web‐based program (Guindon et al., 2010) with 1,000 bootstrap replications and automatic model selection. Visualization of the inferred phylogeny was conducted within the program Dendroscope v3.2.10 (Huson & Scornavacca, 2012).

### 2.6 PCR‐RFLP analysis of all five *Helicoverpa* pest species

Two previous studies have reported methods for distinguishing between *Helicoverpa* species through interrogation of mitochondrial markers, though each was limited in scope. Behere et al. (2008) utilized two restriction enzymes to differentiate *H.* *punctigera*, *H.* *armigera*, *H.* *assulta,* and *H.* *zea* based on RFLPs from the 3′ region of the mtDNA COI gene (511 bp) and a partial Cyt *b* gene region (434 bp), but the study lacked the South American *H.* *gelotopoeon* species that is also a polyphagous pest. The second study, that of Arneodo et al. (2015), used a single restriction enzyme (HinfI) on the 5′ end of the partial COI gene (812 bp) to generate specific RFLP patterns for *H.* *armigera*, *H.* *zea,* and *H.* *gelotopoeon*. Mitogenome resources from this study enabled surveys of the relevant gene regions of *H.* *gelotopoeon*, subspecies of *H.* *assulta* *assulta *and *afra*, *H.* *armigera conferata *and *armigera*, and *H.* *punctigera*, to provide RFLP patterns for all five *Helicoverpa* pest species, thereby improving on the previous work (Arneodo et al., 2015; Behere et al., 2008).

To revise the study of Behere et al. (2008), we analyzed partial mtDNA gene regions (i.e., 3′ region of COI genes of *H.* *gelotopoeon,* trimmed to 511 bp; 5′ region of Cyt *b* gene, trimmed to 411 bp). To update the method of Arneodo et al. (2015), we analyzed the 5′ COI gene regions of *H.* *punctigera* and *H.* *assulta*. Sequences were identified for restriction enzyme(s) and predicted RFLP patterns using the CLC Sequence Viewer 7 program. Relevant partial COI and Cyt *b* gene regions previously reported (Arnemann et al., 2016; Arneodo et al., 2015; Behere et al., 2008; Leite et al., 2016; Tay, Walsh et al., 2017) were also included in the current study (Supporting Information Data S2 and S3—GenBank accession numbers and sequences used for RFLP).

## 3 RESULTS

### 3.1 Molecular characterization of the mitogenomes of *Helicoverpa* (sub)species

The assembled mitogenomes of the five *Helicoverpa *species were estimated to be between 15,226 bp and 15,403 bp in length (Table 1). We identified all 13 PCG's, two rRNA genes, and 22 tRNA genes for all assembled mitogenomes. Perfect synteny was observed between mitogenomes of *H.* *gelotopoeon*, both subspecies of *H.* *assulta,* and previously published *Helicoverpa* species mitogenomes (i.e., Perera et al., 2016; Walsh, 2016; Yin et al., 2010). Intraspecific nucleotide sequence identities were generally low ranging between 93.03% (between *H.* *punctigera* KF977797 and MG437197) and 94.83% (between *H.* *armigera* GU188273 and *H.* *assulta* KT626655), while between the two most recently diverged species *H.* *armigera* and *H.* *zea* this ranged between 96.90% and 97.40% (average = 97.24%). The average intraspecific nucleotide sequence identities were high at the mitogenome level, (i.e., 99.83%, *H.* *punctigera*; 99.67%, *H.* *gelotopoeon*; 98.88%, *H.* *assulta* (excluding chimeric and Sanger sequencing error individuals, see below); 99.46%, *H.* *armigera*; and 99.51%, *H.* *zea*).

Comparing the *H.* *assulta* mitogenomes from this study (MG437197, MG437198, KT626655) to the published (KP015198, Li et al., 2016) and the unpublished (KR149448) *H.* *assulta* mitogenomes highlighted some issues (Table 2). While both *H.* *assulta* mitogenomes assembled in this study were >99% identical, they shared 98% sequence identity with the unpublished *H.* *assulta* mitogenome (KR149448), and only 95% sequence identity with the published *H.* *assulta* mitogenome (KP015198, Li et al., 2016). Similarly, comparison across the 13 PCG's and the two rRNA genes (Table 2) showed that the *H.* *assulta* (KP015198) and the *H.* *armigera* (GU188273) mitogenomes were highly similar, sharing >99%–100% nucleotide identity. For the unpublished *H.* *assulta* mitogenome (KR149448), the lower level of nucleotide sequence identity (98%) with our *H.* *assulta* mitogenomes was predominantly due to low identity from approximately Cyt *b* gene to the rrnS (12S rRNA) gene region (ca. 3,868 bp). Instead, this region of KP015198 was most similar to the *H.* *armigera* (GU188273) individual (Table 2). Because Sanger sequencing for KP015198 was used requiring multiple PCR amplicons across over lapping mitogenome regions, we examined the SNP patterns and ascertained potential regions where contaminations might have occurred from 10,895 (part of Cyt *b*; Table 3) to 14,762 (part of rrnS; Table 4). For all mitogenomes generated in this study, corresponding partial COI genes were also found to match the partial mtCOI genes used in the study of (KP015198, Li et al., 2016; i.e., *H.* *armigera*: EU768935, EU768936; *H.* *assulta*: EU768937; *H.* *gelotopoeon*: EU768938; *H.* *punctigera*: EU768941; *H.* *zea*: EU768942), thereby provided further confirmation of species identity for the mitogenomes presented.

**Table 2:** Nucleotide sequence identity between *Helicoverpa armigera* (GU188273), suspected misidentified *H.* *assulta* (KP0151198), and chimeric *H*. *assulta*/*H.* *armigera* (KR149448), and two *H.* *assulta* *assulta* individuals characterized in this study (MG437197, KT626655) across the 13 protein‐coding genes, and the rrnL (=16S rRNA) and rrnS (=12S rRNA) genes

	GU188273														
	COI (%)	COII (%)	ATP8 (%)	ATP6 (%)	COIII (%)	ND3 (%)	ND5 (%)	ND4 (%)	ND4L (%)	ND6 (%)	COB (%)	ND1 (%)	rrnL (%)	rrnS (%)	ND2 (%)
KP015198	99.74	100	100	100	99.75	100	99.94	99.79	98.13	99.62	99.91	99.78	99.07	99.62	99.79
KR149448	95.89	95.68	96.30	94.99	94.80	91.59	95.81	96.34	95.51	92.57	97.70	99.56	98.64	98.99	96.19
MG437197	95.89	95.68	96.30	94.99	94.80	91.30	95.75	96.34	95.51	92.57	94.15	94.54	91.95	96.25	96.19
KT626655	95.89	95.54	96.30	94.99	94.80	91.30	95.87	96.48	95.51	92.57	94.15	94.54	93.96	96.25	96.19

Note

Shaded regions indicate higher than expected nucleotide sequence identity at the interspecific level between *H.* *armigera* (GU188273) and the presumed *H.* *assulta* (KP015198; KR149448).

**Table 3:** SNP alignment at the mtDNA Cyt *b* gene between *Helicoverpa armigera* (GU188273), two putative *H.* *assulta* individuals (KP015198, KR149448), and two *H.* *assulta* (MG437197; KT626655.1) as reported in this study

					1	1	1	1	1	1	1	2	2	2	2	2	2	2	2	2	3	3	3	3	4	4	4	4	5	5	5	5	5	5	6
		1	3	3	1	3	3	5	7	8	9	1	2	3	4	5	6	7	7	7	0	3	5	6	5	6	9	9	0	3	7	7	9	9	1
	3	8	7	8	2	5	6	6	2	9	5	6	3	4	6	8	7	1	3	7	9	2	5	9	0	2	5	8	7	4	6	9	1	4	2
GU188273	C	C	A	A	T	C	T	C	T	A	T	C	T	C	C	C	C	G	A	T	T	T	C	A	C	T	C	A	C	T	T	T	T	C	C
KP015198	.	.	.	.	.	.	.	.	.	.	.	.	.	.	.	.	.	.	.	.	.	.	.	.	.	.	.	.	.	.	.	.	.	.	.
KR149448	T	T	G	G	C	A	C	T	A	T	C	T	C	T	T	T	T	A	T	A	A	C	.	.	.	.	.	.	.	.	.	.	.	.	.
MG437197	T	T	G	G	C	A	C	T	A	.	C	T	C	T	T	T	T	A	T	A	A	C	T	T	T	G	T	G	T	C	C	C	C	T	T
KT626655	T	T	G	G	C	A	C	T	A	.	C	T	C	T	T	T	T	A	T	A	A	C	T	T	T	G	T	G	T	C	C	C	C	T	T

																									1	1	1	1	1	1	1	1	1	1
	6	6	6	6	6	6	6	7	7	7	7	7	7	7	7	7	7	7	7	8	8	8	9	9	0	0	0	0	0	0	0	0	1	1
	1	2	5	6	6	7	7	0	2	2	3	5	5	6	7	8	8	8	9	4	7	8	4	5	1	2	2	3	4	5	8	9	0	1
	5	1	1	4	5	5	6	2	0	9	9	0	6	2	4	0	3	6	2	6	4	5	5	1	1	4	6	9	0	9	6	6	4	3
GU188273	C	T	C	G	T	C	T	T	T	C	T	A	T	C	A	A	T	T	C	T	G	T	C	T	C	G	C	G	T	C	C	G	C	T
KP015198	.	.	.	.	.	.	.	.	.	.	.	.	.	.	.	.	.	.	.	A	.	.	.	.	.	.	.	.	.	.	.	.	.	.
KR149448	.	.	T	.	.	.	.	.	.	.	.	.	.	T	.	.	.	.	.	A	.	.	.	C	.	.	.	.	.	.	.	.	.	.
MG437197	T	A	T	A	C	T	C	C	C	T	C	T	C	T	T	T	A	A	T	.	A	C	T	.	T	A	T	A	C	T	T	A	T	C
KT626655	T	A	T	A	C	T	C	C	C	T	C	T	C	T	T	T	A	A	T	.	A	C	T	.	T	A	T	A	C	T	T	A	T	C

Note

Nucleotide positions of detected SNPs are indicated (the start of the Cyt *b* CDS marks position 1). SNPs are compared to the GU188273 *H.* *armigera* and identical polymorphisms are indicated by ‘.’. The orange shaded cell at nucleotide position 355 marks the first detectable change over of the *H.* *assulta* SNP pattern for KR149448 to one showing high homology with *H.* *armigera*.

**Table 4:** SNP alignment for the rrnS gene between *Helicoverpa armigera* and the putative *H.* *assulta* individuals (KP015198, KR149448, I.D.343; KT626655.1)

									1	1	1	1	1	2	2	2	2	3	4	4	4	5	5	5	5	6	7	7	7
	6	7	7	7	7	7	9	9	1	6	7	9	9	5	6	6	8	0	2	2	8	1	2	2	4	8	6	7	9
	8	5	6	7	8	9	3	4	9	4	5	1	2	5	4	6	7	3	4	5	5	1	1	4	4	4	5	8	1
GU188273	–	–	–	–	–	–	A	C	T	C	A	G	G	A	C	A	T	T	A	T	T	–	A	T	C	T	T	A	G
KP015198	–	–	–	–	–	–	G	T	G	.	.	.	.	.	.	.	.	.	.	.	.	–	.	.	.	.	.	.	.
KR149448	–	–	–	–	–	–	.	.	.	.	G	.	.	.	.	.	.	.	.	.	A	A	.	A	.	A	C	T	A
MG437197	C	T	T	A	T	T	.	.	.	T	.	C	A	G	A	T	C	C	C	C	A	A	T	A	T	A	C	T	A
KT626655	C	T	T	A	T	T	.	.	.	T	.	C	A	G	A	T	C	C	C	C	A	A	T	A	T	A	C	T	A

Note

Gaps inserted during alignment are indicated by ‘–‘, and ‘.’ indicate nucleotides identical to those in the *H.* *armigera* rrnS gene. The cell highlighted in orange at nucleotide position 425 represents the last identical nucleotide position between KR149448 *H.* *assulta* and *H.* *armigera*. The sequence reverts back to *H.* *assulta* rrnS sequence between positions 426 and 485 predominantly sharing SNPs with Hass‐343 and Hass‐OP from then on. The putative *H.* *assulta* individual KP015198 shares the majority of SNPs with the *H.* *armigera* GU188273, making it very likely that a *H.* *armigera* specimen was misidentified as *H.* *assulta*.

### 3.2 Phylogenetic analysis

**Figure 1:**
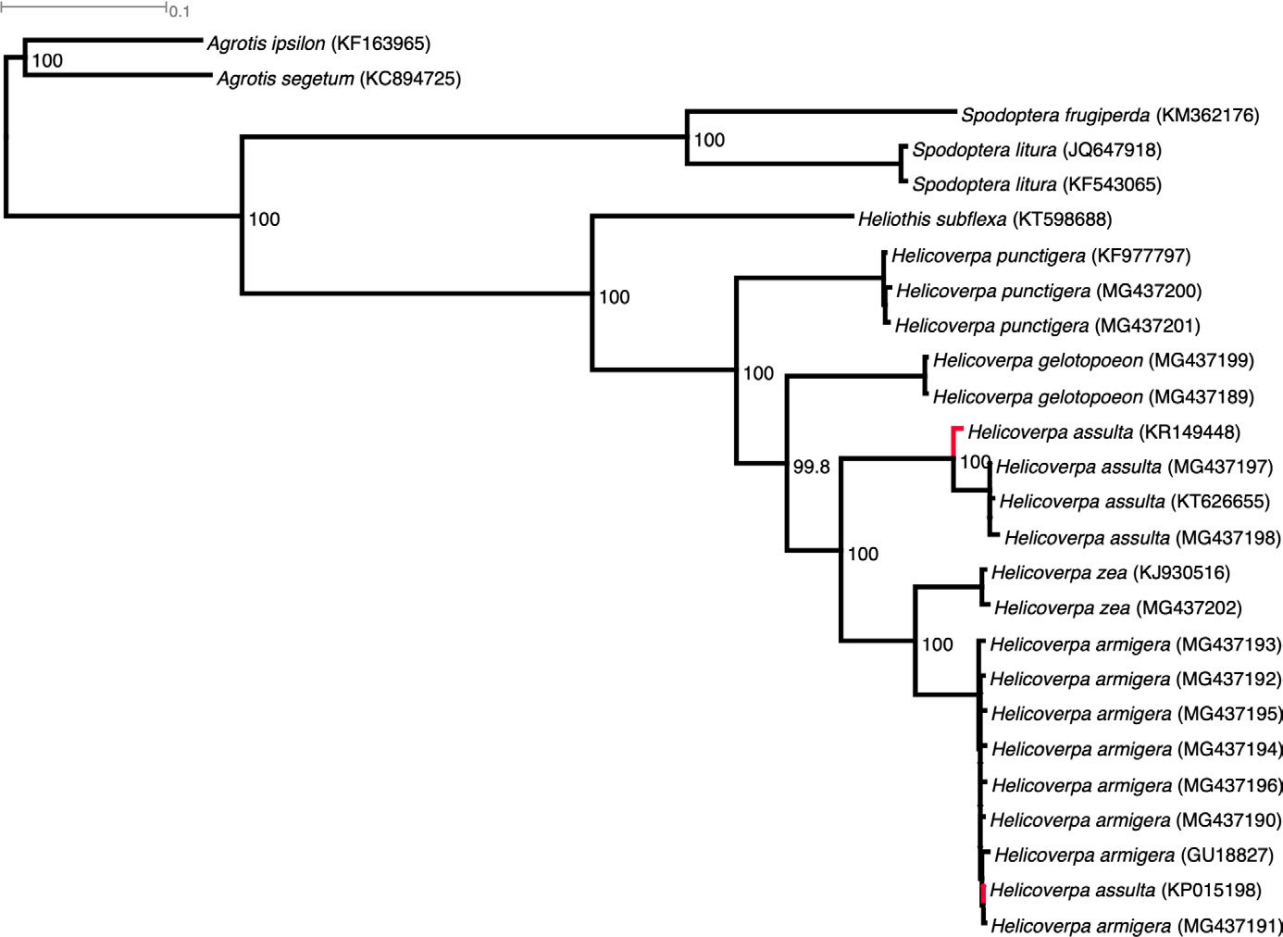
Phylogeny of *Helicoverpa* species from the Old World (*Helicoverpa armigera*, *H.* *assulta*), Australia (*H.* *punctigera*), and the New World (*H.* *zea*, *H.* *gelotopoeon*) and related noctuid species (*Heliothis* (*Chloridea*) *subflexa*; *Spodoptera litura*, *S.* *frugiperda*, *Agrotis ipsilon*) based on 13 aligned protein‐coding gene regions (10,310 bp) from mitochondrial genomes. Phylogenetic analysis was carried out using the PhyML. Branches shown in red identify misidentified and chimerical *H.* *assulta *mitochondrial genomes deposited in GenBank

PhyML identified the optimal nucleotide evolutionary model as GTR+G+I+F (InL ‐33794.41; AIC 71706.82; BIC 72137.99). Phylogenetic analysis using the GTR substitution model, 0.616 proportion of invariable sites, four substitution rate categories and an estimated 1.140 gamma‐shape parameter with 1,000 bootstraps was performed. The phylogeny (Figure 1) is based on the trimmed 13 PCG's and includes as outgroups *A.* *segetum* (KC894725), *A.* *ipsilon *(KF163965),* S.* *frugiperda* (KM362176),* S.* *litura* (KF543065, JQ647918), and *Chloridea* (*Heliothis*) *subflexa,* resulting in *H.* *punctigera* being sister to all other Helicoverpa species, namely *H.* *gelotopoeon*, *H.* *assulta*, and with *H.* *zea* and *H.* *armigera *as the most recently diverged sister clades. As expected, the *H.* *assulta* mitogenome with partial *H.* *armigera* contamination (i.e., KR149448) was sister to the two subspecies of *H.* *assulta*. Similarly, the published *H.* *assulta* (KP015198) clustered within the global representation of *H.* *armigera* (i.e., *H.* *armigera* *armigera* and *H.* *armigera* *conferta*). Subspecies status (i.e., within *H.* *assulta*, and within *H.* *armigera*) could not be differentiated based on mitochondrial DNA genomes. All nodes had high (99.8%–100%) support values, with 100% bootstrap values obtained for each of the *Helicoverpa* species. The phylogenetic relationships between the pest *Helicoverpa* species were well‐resolved using the complete mitogenome protein‐coding gene sequences of 10,310 bp (Supporting Information Data S1), with the same tree topology as previously reported by (Anderson et al., 2016). The phylogenetic relationship between *H.* *zea* and *H.* *armigera* suggested these two species shared a most recent common ancestor that diverged approximately 1.5–2 million years ago (Behere et al., 2007; Pearce et al.2017a, 2017b). The monophyly of each of the subspecies of *H.* *assulta* (i.e., *H.* *assulta* *assulta*, *H.* *assulta* *afra*) and of *H.* *armigera* (i.e., *H.* *armigera* *armigera*, *H.* *armigera* *conferta*) was not well‐resolved, with low bootstrap values at internal nodes (Figure 1). However, Anderson et al. (2016) was able to differentiate Australian *H.* *armigera* *conferta* from Old World *H.* *armigera* *armigera* based on genome‐wide SNP data. A similar genome‐wide SNP approach might be used to differentiate subspecies of *H.* *assulta*.

### 3.3 RFLP analysis

The PCR‐RFLP method of Behere et al. (2008) and Arneodo et al. (2015) that used 511 bp of the c‐terminal COI gene, and 698 bp (trimmed from 732 bp) from the N‐terminal COI gene, respectively, were revised such that either the N‐terminal or C‐terminal region of partial COI gene could be used to differentiate all five pest *Helicoverpa* species through a combination of four restriction enzymes (Table 5). The restriction enzymes selected represented a more comprehensive in silico analysis of publicly available partial COI sequences and avoided the need to include the partial Cyt *b* gene RFLP analysis originally designed by Behere et al. (2008). The revised RFLP method has not included the restriction enzymes identified by both Arneodo et al. (2015) and Behere et al. (2008) because of novel mtCOI haplotypes identified from global populations of *H.* *armigera* including from South America (e.g., Arnemann, 2015; Arnemann et al., 2016; Leite et al., 2016; Tay, Walsh et al., 2017).

**Table 5:** Predicted PCR‐RFLP patterns on 698 bp (5′ region) and 511 bp (3′ region) partial COI gene regions of the five *Helicoverpa* species

*Helicoverpa* species	COI (698 bp) Arneodo et al. (2015)			*BpuEI*	COI (511 bp)			
	*BseJI*	*Bsa*I	*Bsa*BI		*Bco5*I /	*AquVI*	*BseR*I	*Eco130I*
*punctigera*	✗1.0.698 (698 bp)	✓1.0.494 (494 bp) 494.0.698 (204 bp)	✗1.0.698 (698 bp)	✗1.0.698 (698 bp)	✗1.0.511 (511 bp)	✗1.0.511 (511 bp)	✓1.0.468 (468 bp) 469.0.511 (43 bp)	✗1.0.511 (511 bp)
*armigera*	✗1.0.698 (698 bp)	✗1.0.698 (698 bp)	✗1.0.698 (698 bp)	✗1.0.698 (698 bp)	✗1.0.511 (511 bp)	✗1.0.511 (511 bp)	✗1.0.511 (511 bp)	✗1.0.511 (511 bp)
*assulta*	✓1.0.461, (461 bp) 461.0.698 (237 bp)	✗1.0.698 (698 bp)	✓1.0.461 (461 bp) 461.0.698 (237 bp)	✗1.0.698 (698 bp)	✗1.0.511 (511 bp)	✓1.0.267 (267 bp) 268.0.511 (244 bp)	✗1.0.511 (511 bp)	✗1.0.511 (511 bp)
*zea*	✗1.0.698 (698 bp)	✗1.0.698 (698 bp)	✗1.0.698 (698 bp)	✓1.0.584 (584 bp) 584.0.698 (114 bp)	✓1.0.96 (96 bp) 97.0.511 (415 bp)	✗1.0.511 (511 bp)	✗1.0.511 (511 bp)	✗1.0.511 (511 bp)
*gelotopoeon*	✗1.0.698 (698 bp)	✗1.0.698 (698 bp)	✗1.0.698 (698 bp)	✗1.0.698 (698 bp)	✗1.0.511 (511 bp)	✓1.0.458 (458 bp) 459.0.511 (53 bp)	✗1.0.511 (511 bp)	✓1.0.300 (300 bp), 300.0.511 (211 bp)

Note

Differentiation between all five *Helicoverpa* pest species for the N‐terminal (698 bp) COI gene region required restriction endonucleases of BseJI, BsaL, BasBI, and BpuEI. For the PCR‐RFLP of the 511 bp C‐terminal region of partial COI gene, Bco5I, AquVI, BseRI, and Eco130I restriction endonucleases were identified. The presence and absence of restriction enzyme cut sites within amplicons are indicated by ✓ and ✗, respectively. Expected restriction fragment lengths from either the 698 bp or 511 bp partial COI gene regions are indicated accordingly.

## 4 DISCUSSION

In this study, we increased the mitogenome resources for the five globally significant agricultural *Helicoverpa* species including subspecies for *H.* *assulta* and *H.* *armigera, *as well the South American endemic *H.* *gelotopoeon*. Furthermore, we report on species identification and mitogenome assembly issues with existing data for the Solanaceae specialist *H.* *assulta*. These mitogenome resources include *H.* *armigera armigera* subspecies from its Old World range as well as its New World invasive range. We also report the complete draft mitogenome of *H.* *zea* from Brazil, where previously only data from the North American continent were available (Perera et al., 2016). Finally, we have revised existing PCR‐RFLP methods to allow for identification between these *Helicoverpa* pest species, taking into consideration the increase in partial mtDNA sequences resources in public DNA database. In addition, this method removes the need to survey the Cytb gene region for confirmation of species, thereby eliminating possible detection failures due to PCR amplification failures for the Cyt *b* gene.

Species identification in the genus *Helicoverpa* can be difficult and potentially led to the delay in recognizing *H.* *armigera* in South America (Tay et al., 2013). Species misidentification may have also resulted in the incorrect reporting of the *H.* *assulta* mitogenome (KP015198, Li et al., 2016). This misidentification is supported by the COI phylogeny (data not shown), by BlastN searches involving the regions of the COI and Cyt *b* gene (e.g., Behere et al., 2008; Behere et al., 2007), and by the concatenated mitogenome PCG's phylogeny (Figure 1). For the second publicly available *H.* *assulta* mitogenome (GenBank KR149448), we demonstrated that this mitogenome was a chimerical assembly that consisted of fragments of *H.* *assulta* and *H.* *armige*ra mitogenomes, with the portion of the mitogenome between the Cyt *b* gene to the rrnS gene likely the result of genomic DNA contamination during PCR.

The incorrectly reported *H.* *assulta* mitogenome (Li et al., 2016) may have arisen as a result of hybridization between a female *H.* *armigera* and a male *H.* *assulta *which may have appeared morphologically similar to *H.* *assulta*. Hybrids resulting from mating between *H.* *armigera* and *H.* *assulta* are known to occur under experimental conditions (e.g., Wang, Zhao, & Wang, 2005; Zhao et al., 2005), though there has been no molecular investigation of the nature of the hybridization. The individual collected and sequenced by Li et al. (2016) originated from a cotton field which is unusual for a Solanaceae specialist such as *H.* *assulta* (Ahn, Badenes‐Perez, & Heckel, 2011), though *H.* *assulta* from cotton fields in Australia has occasionally also been reported (Sharon Downes and Mary Whitehouse CSIRO, pers. comm.). Hybridization is also possible between *H.* *armigera* and *H.* *zea*, for example, Hardwick (1965), Laster and Hardee (1995), and Laster and Sheng (1995) have shown that under laboratory conditions, *H.* *armigera* can mate and produce fertile offspring with *H.* *zea*. The possibility of natural hybridization between *H.* *armigera* and *H.* *gelotopoeon* is also unknown, and with the recent incursions of *H.* *armigera* into the New World, potential interspecific hybridization between *H.* *armigera* and *H.* *gelotopoeon* will need to be investigated. Should any hybridization occur, mitochondrial markers can only identify the matriline and the mitochondrial DNA PCR‐RFLP method will not be able to identify these hybrids. However, *H.* *armigera‐H. zea* hybrids can be detected using genome‐wide SNPs based on high‐throughput sequencing methods (Anderson et al., 2018).

Microsatellite DNA markers in the Lepidoptera including *Helicoverpa* species are often affected by transposable elements (Gordon, Tay, Collinge, Williams, & Batterham, 2010; Tay, Behere, Batterham, & Heckel, 2010), and alternative nuclear DNA markers should be used instead where possible (i.e., EPIC‐PCR markers, see Behere, Tay, Russell, Kranthi, and Batterham (2013) and Tay, Behere, Heckel, Lee, and Batterham (2008). Nevertheless, signatures of interspecific *H.* *armigera*‐*H.* *zea* hybridization as inferred from microsatellite loci have been reported by Leite et al. (2016). Future monitoring of *Helicoverpa* species at preborder inspection could also consider the method of Nagoshi, Gilligan, and Brambila (2016) that incorporates the z‐linked Triosephosphate isomerase (Tpi) gene with the mtCOI gene for identifying potential *H.* *armigera*‐*H.* *zea* hybrids, although the feasibility of transferring this method to screen for potential *H.* *armigera*‐*H.* *gelotopoeon*,* H.* *assulta*‐*H.* *gelotopoeon*, *H.* *assulta*‐*H.* *zea*, and *H.* *armigera*‐*H.* *assulta* hybrids remained to be tested. Failure to monitor for these interspecific hybrids may lead to invasive genotypes such as enhanced resistance to insecticides being spread unchecked (Walsh et al., 2018). The identification of naturally occurring hybrids will be difficult and will require significant coordination efforts between governmental departments (e.g., quarantine services, molecular detection, and identification facilities), and the development and adoption of new biosecurity policies. However, most of these are not yet recognized by policy makers as novel, potential and/or imminent national biosecurity threats.

Regardless of the shortcomings of mitochondrial genes in assisting with species confirmation, it is nevertheless desirable to obtain well‐characterized mtDNA genes to bolster biosecurity and pest management practices. The importance of rechecking the assembled mtDNA against public DNA databases (e.g., NCBI Genbank, BoLD) is often not emphasized and has on occasion, led to the misidentification of species and mitogenomes (Tay, Elfekih, Court, Gordon, & Barro, 2016; Walsh, 2016). While providing the much needed mitogenome resources for the *Helicoverpa* pest species across the Old and New Worlds, our study is not aimed at criticizing mistakes and oversights, but is rather, a cautionary reminder of the need to check sequence data against that readily available public DNA databases.

## CONFLICT OF INTEREST

None declared.

## AUTHOR CONTRIBUTIONS

WTT, TW, KG, and AM conceived and designed the study. WTT, TW, OP, CA, and AM contributed to the work. All the authors contributed material, data, analysis and to the writing of the manuscript.

## Data Availability

All assembled mitogenomes are available in NCBI Genbank (Table 1). Raw data is available from the CSIRO data portal (https://doi.org/10.4225/08/5ab8fd3de72d3).
